# Bacteriophages with the Ability to Degrade Uropathogenic *Escherichia Coli *Biofilms

**DOI:** 10.3390/v4040471

**Published:** 2012-04-10

**Authors:** Andrew Chibeu, Erika J. Lingohr, Luke Masson, Amee Manges, Josée Harel, Hans-W. Ackermann, Andrew M. Kropinski, Patrick Boerlin

**Affiliations:** 1 Laboratory for Foodborne Zoonoses, Public Health Agency of Canada, Guelph, ON, N1G 3W4, Canada; Email: erika.lingohr@phac-aspc.gc.ca; 2 Biotechnology Research Institute, National Research Council of Canada, 6100 Royalmount Avenue, Montréal, QC, H4P 2R2, Canada; Email: luke.masson@cnrc-nrc.gc.ca; 3 Département de microbiologie et immunologie, Université de Montréal, 2900, boul. Édouard-Montpetit, Montréal, QC, H3T 1J4, Canada; 4 Department of Epidemiology, Biostatistics and Occupational Health, McGill University, 1020 avenue des Pins Ouest, Montréal, QC, H3A 1A2, Canada; Email: amee.manges@mcgill.ca; 5 Groupe de Recherche sur les Maladies Infectieuses du Porc (GREMIP) and Centre de Recherche en infectiologie porcine (CRIP), Université de Montréal, Faculté de médecine vétérinaire, Saint-Hyacinthe, QC, J2S 7C6, Canada; Email: josee.harel@umontreal.ca; 6 Felix d’Herelle Reference Center for Bacterial Viruses, Department of Microbiology, Immunology and Infectionlogy, Faculty of Medicine, Laval University, QC, G1K 4C6, Canada; Email: ackermann@mcb.ulaval.ca; 7 Department of Molecular and Cellular Biology, University of Guelph, ON, N1G 2W1, Canada; 8 Department of Pathobiology, Ontario Veterinary College, University of Guelph, ON, N1G 2W1, Canada; Email: pboerlin@uoguelph.ca

**Keywords:** UPEC, bacteriophage, biofilms

## Abstract

*Escherichia coli*-associated urinary tract infections (UTIs) are among the most common bacterial infections in humans. UTIs are usually managed with antibiotic therapy, but over the years, antibiotic-resistant strains of uropathogenic *E. coli *(UPEC) have emerged. The formation of biofilms further complicates the treatment of these infections by making them resistant to killing by the host immune system as well as by antibiotics. This has encouraged research into therapy using bacteriophages (phages) as a supplement or substitute for antibiotics. In this study we characterized 253 UPEC in terms of their biofilm-forming capabilities, serotype, and antimicrobial resistance. Three phages were then isolated (vB_EcoP_ACG-C91, vB_EcoM_ACG-C40 and vB_EcoS_ACG-M12) which were able to lyse 80.5% of a subset (42) of the UPEC strains able to form biofilms. Correlation was established between phage sensitivity and specific serotypes of the UPEC strains. The phages’ genome sequences were determined and resulted in classification of vB_EcoP_ACG-C91 as a *SP6likevirus*, vB_EcoM_ACG-C40 as a *T4likevirus* and vB_EcoS_ACG-M12 as *T1likevirus*. We assessed the ability of the three phages to eradicate the established biofilm of one of the UPEC strains used in the study. All phages significantly reduced the biofilm within 2–12 h of incubation.

## 1. Introduction

Urinary tract infections (UTIs) are among the most common bacterial infections in humans. They account for more than seven million visits to physicians’ offices per year in the United States of America [1]. Approximately 20% of women develop UTIs sometime during their lifetime. Above the age of 50, men and women have a similar incidence of UTIs [1,2,3,4,5]. UTIs have tremendous economic impact on health care systems in both direct and indirect costs associated with treatment [6,7].

Uropathogenic strains of *Escherichia coli* (UPECs) account for about 75–85% of UTIs [4,8]. There has been an evolution toward antibiotic resistance in UPECs, with decreasing susceptibility to first-line agents such as ampicillin, nitrofurantoin, sulphamethoxazole/trimethoprim (SXT) and fluoroquinolones [4,9,10,11,12,13]. An alternative to antimicrobial treatment would be of great relevance to treat multiresistant UTIs [5], as well as to avoid the continuous selection of resistant pathogens and to safeguard the efficacy of antimicrobial agents for cases of emergency and life-threatening infections.

In hospital-acquired infections as well as in geriatrics patients, most UTI are associated with indwelling catheters which act as foci for biofilm formation [14]. Biofilms play a significant role in the ability of bacteria to withstand killing by host immune responses and antibiotics. There is a need to develop new therapeutic strategies to eradicate biofilm infections [15].

The development of biofilms by bacteria does not protect cells from bacteriophage killing. These viruses can penetrate the extracellular matrix that binds macromolecules and prevents their diffusion into the biofilm [16,17,18] and kill cells [19,20,21]. Furthermore, certain phages have evolved to deal with capsules by possessing virion-associated polysaccharide depolymerases [22,23,24] leading us to believe that they are ideal agents to help reduce biofilm-associated infections as well as to kill planktonic cells.

In this paper, we describe a subset of a collection of 253 *E. coli *isolates from UTIs specifically selected on their ability to form biofilms and the activity of three environmentally isolated bacteriophages on these strains. To assess their efficacy and safety as therapeutic agents against UPEC, the phages were characterized in detail with regards to their spectrum of activity, genetic structure, as well as activity on biofilms. 

## 2. Results and Discussion

### 2.1. Biofilm Forming Capabilities, Serotype and Antimicrobial Resistance

**Figure 1:**
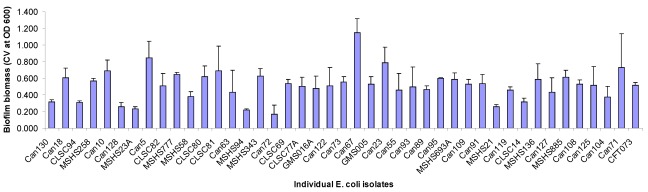
Urinary tract infection (UTI) *E. coli* isolates positive for biofilm formation on 96-well microtiter plates. Isolates were considered as biofilm formers if the OD_600_ for the crystal violet-stained biofilm was equal to or greater than 3-fold the OD_600 _for a bacteria free medium. Data points represent an average of three independent experiments, each with 6 replicate wells for each isolate tested. Isolate CTF073 is a positive control for biofilm formation. Error bars indicate standard error of means.

Each of the 253 UTI *E. coli *isolates was screened for static biofilm formation in 96-well untreated polystyrene microtiter plates following growth in artificial urine medium as described in the Experimental section. Isolates were considered positive for biofilm formation if the crystal violet-stained biofilm had an OD_600_ equal to or greater than 3-fold the value obtained in the well containing bacteria-free medium. The UTI *E. coli *strain CTF073 was used as a positive control and 12 wells of this strain were included with each plate of samples of UPEC assayed for biofilm formation. The plotted results were the average of three independent experiments, each with six replicates per isolate. Only 42 out of the 253 *E. coli *isolates tested in this study produced biofilms in microtiter plate wells with the levels of biofilm formation varying among isolates (Figure 1). This is in contrast to a previous study [25] where a majority of the UTI strains tested produced biofilms *in vitro*. A possible reason for the different outcomes in the two studies is the fact that UPEC do not form biofilm well on polystyrene plates in urine or artificial urine medium as compared to M9 minimal medium used by [25]. Among the 42 biofilm-forming isolates, Can67 showed the highest level of biofilm formation (OD_600_ above 1.0) whereas MSHS94 and Can72 resulted in weak biofilms formation with an OD_600_ below 0.2.

The biofilm-forming UTI *E. coli *isolates totalized 22 different somatic O antigens and 14 different H antigens (Table 1). We then investigated their susceptibility profiles against 15 antimicrobials. The investigated isolates were frequently resistant with 32 (78%) isolates displaying resistance to antimicrobial agents, while 16 (39%) isolates displayed multidrug resistance phenotypes. These results are in agreement with previous studies that have shown a high frequency of antimicrobial resistance among UPEC [4,9,10,11,12,13]. However, inspection of the different levels of biofilm formation on microtiter plates (Figure 1) and comparing this to the results on antimicrobial susceptibility (Table 1) showed that there was no apparent direct correlation between antimicrobial resistance and the amount of biofilm formed by each isolate.

**Table 1:** List of UTI *E. coli *forming biofilms, their serotypes, respective antimicrobials resistances and sensitivity to the three isolated phages ACG-C91, ACG-C40 and ACG-M12. AMC = Amoxicillin/Clavulanic acid; AMP = Ampicillin; FOX = Cefoxitin; TIO = Ceftiofur; CRO = Ceftriaxone; CHL = Chloramphinicol; CIP = Ciprofloxacin; KAN = Kanamycin; NAL = Nalidixic acid; STR = Streptomycin; SOX = Sulfisoxazole, TCY = Tetracycline; SXT = Sulphamethoxazole/Trimethoprim. + symbolises sensitivity to phage; - resistance to phage; nd, not determined.

Strain	Serotype	Antimicrobials to which isolates are resistant	ACG-C40	ACG-C91	ACG-M12
Can130	O1:NM		+	+	-
Can18	O2:H4		-	+	-
CLSC94	O2:H7		+	+	-
MSHS258	O2:H18		+	+	-
Can10	O8:H10		-	-	-
Can128	O8:NM	SOX, TCY	+	-	-
MSHS23A	O6:H1	AMP, STR, SOX,	+	-	+
Can5	O6:H1	NAL	+	-	+
CLSC82	O6:H1	AMP	+	-	+
MSHS777	O6:H1	STR	+	-	+
MSHS58	O6:H25	KAN	-	-	-
CLSC80	O11:H18	STR, SOX	+	-	-
CLSC81	O14:H4		+	-	+
Can63	O14:H31	TCY	-	-	+
MSHS94	O18ac:H7	AMP, STR, SOX, TCY	+	+	+
MSHS343	O18ac:H7		+	+	+
Can72	O18ac:NM	AMP	+	-	-
CLSC69	O21:H14		-	-	-
CLSC77A	O22:H1		-	-	-
GMS016A	O25:H1		+	+	+
Can122	O25:H4	AMP, TIO, CRO, CIP, NAL, SOX, TCY, SXT	-	-	-
Can73	O25:H4	AMP, CIP, NAL	-	-	-
Can67	O25:H4	AMC, AMP, CIP, NAL	-	-	-
GMS005A	O35:H10		+	-	-
Can23	O68:H18		+	-	-
Can55	O75:H7	AMC, AMP, FOX, TIO, CRO	+	+	-
Can93	O75:H7		-	+	-
Can89	O78:H5		+	-	-
Can95	O106:H18	CHL, TCY	-	-	-
MSHS693A	O117:H5		+	-	-
Can109	O134:H31	NAL	+	+	-
Can91	O135:H6		+	+	+
MSHS21	O135:H11		+	-	-
Can119	O153:H18	AMP, TIO, CRO, NAL, STR, SOX, SXT	+	+	-
CLSC14	O153:NM	AMP, STR, SOX, TCY, SXT	+	-	-
MSHS136	O166:H15	AMC, AMP	-	-	-
Can127	OR:H4	AMP, CHL, TCY	-	+	+
MSHS885	OR:H4	AMP, CHL, STR, SOX, TCY	+	+	-
Can108	OR:H4	SOX, TCY, STX	+	+	-
Can125	OR:H4	AMP, CIP, NAL, SOX, SXT	+	-	+
Can104	OR:H7		-	+	+
Can71	OR:H40		+	-	+
CFT073	nd	nd	+	+	+

It is important to note that the ability of biofilms to withstand antibiotic killing is a function of their mode of growth. Antimicrobial agents have been shown to penetrate biofilms at different rates depending on the particular agent and the biofilm [26]. The antimicrobial susceptibility tests carried out in this study using the broth microdilution method did not test the susceptibility of the bacteria to the antimicrobial agent after biofilm formation. The antimicrobials were added to cell suspensions at time of media inoculation and not after the biofilms had formed. The results of [27] demonstrated that older biofilms of *E. coli *resisted ampicillin treatment to a greater extent than their younger counterparts. However, in our study, a majority of the biofilm forming *E. coli *UTI isolates (78%) were resistant to at least one antimicrobial agent. This compares to 53.1% of the non-biofilm forming *E. coli* UTI isolates that were resistant to at least one out of the 15 antimicrobials tested (results not shown). This is consistent with what is known about antimicrobial resistance among biofilm forming bacterial strains [28].

### 2.2. Phage Morphology

UPEC strains Can 91, Can 40 and MSHS1210 were used as propagating strains for the isolation of phages from serially diluted CsCl purified phage concentrate. All three isolates were from midstream urine specimens with Can 40 and Can 91 being part of the CANWARD study whereas MSHS1210 was from the McGill University student health service. The single unique phages isolated from UPEC strains Can 91, Can 40 and MSHS1210 were vB_EcoP_ACG-C91, vB_EcoM_ACG-C40 and vB_EcoS_ACG-M12, respectively.

**Figure 2:**
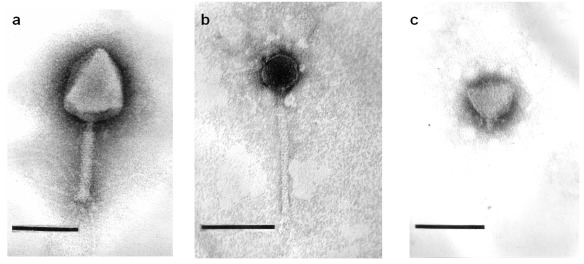
Negative staining of phages ACG-C91 (**a**); ACG-M12 (**b**) and ACG-C40 (**c**) with 2% uranyl acetate or 2% phosphotungstate. Final magnification is × 297,000. Bars indicate 100 nm.

Transmission electron microscopy revealed that phage ACG-C40 has an elongated head, a neck, and a contractile tail with tail fibers (Figure 2a). Its head is 110 × 82 nm, while the extended tail is 114 × 8 nm. Phage ACG-M12 (Figure 2b) has an isometric head of about 157 nm in diameter between opposite apices and a relatively flexible tail of 172 × 7 nm, which terminate in 1–2 fibers of 12 nm in length. Phage ACG-C91 (Figure 2c) has an isometric head of 65–68 nm and a short tail of 12 × 8 nm, which carries fibers of 13 nm in length with a terminal swelling. Based on their morphology, ACG-C40 is classified as a member of the family *Myoviridae*, ACG-M12 is part of the *Siphoviridae *and ACG-C91 belongs to the family *Podoviridae*. 

### 2.3. Lytic Spectra of Phages against Biofilm Forming UTI Isolates and Relation to Isolates’ Serotypes

The lytic activity of ACG-C91, ACG-C40 and ACG-M12 tested against the biofilm forming *E. coli *UTI isolates. In the spot tests, 33 (80.5%) of the biofilm forming *E. coli *UTI isolates were infected by at least one of the three phages (Table 1). 

Out of the three phages isolated in this study, phage ACG-C40 had the broadest host range, with the ability to lyse 28 (66.67%) of the biofilm forming UTI *E. coli *isolates tested (Table 1). It is not unusual for phages in the family *Myoviridae *to have such a large host range. A previous study by [29], showed that bacteriophage T4 lysed 41.1% of the 69 clinical isolates of *E. coli *tested. Other T4-like phages such as AR1 have been observed to have similar broad host ranges [30]. The ability of phage ACG-C40 to cause lysis in a wide range of *E. coli *isolates with different serotypes makes it an important candidate for phage therapy applications. The broad host range of UTI *E. coli *isolates is an indicator of how well adapted phage ACG-C40 is to infecting UPEC, which in itself is a desirable property for a phage to be used in phage therapy against such strains. The criteria of choosing phages which are well adapted for infection of targeted hosts for therapeutic purposes have been applied in similar previous studies [31,32,33]. 

The correlation between host serotype and phage sensitivity is seen in the fact that among the biofilm forming strains, all the O25:H4 strains were resistant to the three tested phages. The O6:H1 strains were sensitive to phages ACG-C40 and ACG-M12. Further in depth studies to identify the receptors of the tested phages and the receptors spatial orientation on the surface of the host strains under tested conditions would give insight as to why the phages are resistant or susceptible to all strains of a given serotype.

### 2.4. Salient Bacteriophage Genome Features

Genome sequencing and bioinformatics analysis of the three isolated phages revealed that all three are non-temperate and that none carries any known bacterial virulence genes for humans or animals. None of the three phages possessed a demonstrable extracellular polysaccharide or exopolysaccharide (EPS) depolymerase gene. The importance of phage-associated depolymerases in biofilm eradication has been recognized in previous studies [22,23,24,34]. Depolymerase-producing phages were found to be more effective in eradicating mature biofilms than non-depolymerase producing phages [34]. Phages producing depolymerases have thus been used in concert with antimicrobials to facilitate deeper penetration of antimicrobials by degrading the EPS [23,34]. However, phages that do not produce EPS depolymerases have also found use in biofilm degradation. Such phages include naturally occurring phages such as T4 [16,17,35] and phages such as T7 engineered to express recombinant dispersin B (DsbB) [36].

#### 2.4.1. Phage ACG-C40

The sequence of the phage genome consisted of 167,396 bp (G+C content 35%) which is close to the genome size of other “T4-like viruses” [37]. It was predicted to encode 282 ORFs and 10 tRNAs. The latter clustered between 68, 043 and 68, 989 bp on the phage’s genome. 

The DNA of this virus was resistant to digestion by all restriction endonucleases tested. An analysis of the genome of this phage (Additional file: Figure A1; Table A1) reveals that it does, like coliphage T4, encode, a glucosyl transferase (orf 060). Bacteriophage T4 contains glucosylated 5-hydroxymethylcytosine (hmdCyt) instead of cytosine which makes its DNA resistant to most restriction enzymes except for EcoRV and NdeI [38]. Based on the presence of the glucosyl transferase gene and the resistance to enzymes, we conclude that bacteriophage ACG-C40 also contains hypermodified bases, probably glucosylated hmdCyt.

The genome of this phage revealed that the phage potentially encodes two versions of the Hoc protein; a shorter and a longer version which may result from translational frameshifting generating Hoc protein with C termini of different lengths (supplementary file: Table S1). 

#### 2.4.2. Phage ACG-C91

Phage ACG-C91 has a 43,731 bp genome with G+C content of 45% and predicted 55 ORFs (supplementary file: Table S2). The phage DNA was sensitive to restriction enzymes BglI, XbaI, EcoRV, EcoRI, NdeI and SalI.

#### 2.4.3. Phage ACG-M12

Phage ACG-M12 has a 46, 054 bp genome with a G+C content of 44% and 77 ORFs (supplementary file: Table S3). The phage DNA was sensitive to all restriction enzymes tested except NaeI and SmaI.

### 2.5. Comparative Genomics

Pairwise comparisons using CoreGenes 3.0 [39] at default stringency setting (“75”) revealed that at the protein level, ACG-C40, ACG-C91 and ACG_M12 were 85.9% ,78.8% and 77.3% similar to bacteriophages T4, SP6 and RTP, respectively. Bacteriophage ACG-C40 genome encodes 239 proteins homologous to T4 proteins, ACG-C91 genome has 41 encoded proteins homologous to those of SP6 and ACG-M12 genome encodes 58 proteins with homology to RTP proteins (supplementary files: Table A1; Table A2; Table A3).

A Mauve alignment [40,41] of phage ACG-C91 DNA against the genome of phage SP6 shows regions of sequence similarity (coloured red) with two regions where there are no DNA sequence similarities (coloured white). The regions of non-sequence similarity correspond to the regions encoding phage ACG-C91 internal virion protein proteins (orf 38), putative tail protein (orf 41) and the endosialidase (orf 53). The genome region encoding the endosialidase is lacking altogether in phage SP6 (supplimentary file: Table A1; supplementary file: Figure A2a). This protein is found in K1-specific phages, such as K1F and K1-5, that encode virion-associated endosialidases hydrolyzing the K1 polysialic acid structure of the K1 capsule-producing *E.coli *strains. Mauve alignment of ACG-M12 against the genome of phage RTP was also performed and it revealed regions of sequence similarity (red) interspersed with four regions where no sequence similarity exists (white). The regions of genome homology disparity include phage ACG-M12 genome regions encoding: hypothetical proteins orf2, orf3 and orf4 (1025 bp–1588 bp), the major tail protein orf26 (12435 bp–13091 bp), putative tail fibre orf47 (29255–32074) and conserved hypothetical proteins orf62 and orf63 (supplementary file: Figure A2 b).

### 2.5. Bacteriophage Eradication of Established Biofilms

**Figure 3:**
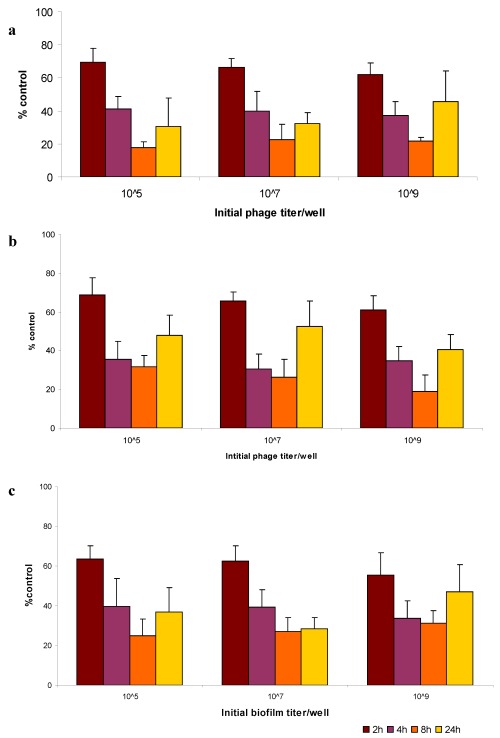
Phage disruption of established *E. coli *strain Can 91 biofilm. Biofilms grown in polystyrene microtiter plate wells for 48 h, were initially inoculated with 10^5^, 10^7^ and 10^9^ pfu of phages (**a**) ACG-C40 and (**b**) ACG-C91 (**c**) ACG-M12. After 2, 4, 8 and 24 h of phage treatment at 37°C, average biofilm biomass in corresponding microtiter plate wells, were scored relatively to untreated control samples (100%) and represented on the Y-axes. Three independent experiments were performed, each starting from a different overnight culture and each with six repeats for each parameter combination. Error bars indicate standard error of means.

UTI* E. coli *isolate Can 91 was selected to determine the effectiveness of the isolated phages to degrade established biofilms because of the isolate’s ability to form strong biofilms in microtiter wells (OD_600_ 0.538) after 48 hrs and its sensitivity to all the three phages isolated. Phages ACG-C40, ACG-C91 and ACG-M12 treatment of preformed UTI* E. coli* Can 91 biofilms yielded reductions (based on OD_600_ measurement of CV-stained cells) in biofilm mass compared to untreated controls (Figure 3). All of the three phages displayed, at low concentrations (10^5^ PFU/mL), a steady reduction of biofilm biomass over time with the largest percentage reduction of biofilm biomass being realized after 8 h incubation. The same pattern was also observed at higher phage concentrations (10^7^ PFU/mL and 10^9^ PFU/mL). At all phage concentrations, it was evident that the biofilm started re-establishing itself after 24 h (Figure 3).

These results indicate that under the conditions tested, the biofilm reduction by the phages is not dose-dependent, a fact that could be taken advantage of if the phages are to be applied *in vivo *to remove established biofilms. It would mean that low titers of the phages were as effective as using a higher titer in eradicating established biofilms. Re-establishment of biofilms after 24 h exposure to the phages *in vitro *may be attributed to development of bacterial resistance against the phages. This could be overcome by using a cocktail of phages. It may also be a good strategy to augment phage treatment with chemical antimicrobials which will be effective in prevention of the re-establishment of biofilms by the phage resistant cells.

Further studies need to be carried out to assess the efficacy of phage ACG-C40, ACG-C91 and ACG-M12 in the prevention of biofilm formation for comparison with this study where we have assayed their efficacy in the eradication of established biofilms. 

## 3. Experimental Section

### 3.1. Sampling and *E. coli* Strain Isolation

A group of 123 strains of UTI *E. coli* were isolated from women with community-acquired UTI either from a student health service (McGill University, Montréal, QC, Canada) or at a community health center also in Montréal. To ensure no bias in the selection of isolates due to recurrent UTI or treatment failures, only one isolate per patient was used. 

An additional 130 isolates were part of the ongoing Canadian national surveillance study (CANWARD) for testing urinary culture pathogens for antimicrobial susceptibilities. The study involved out-patients attending hospital clinics and emergency rooms, and in patients on medical and surgical wards, and in intensive care units [42]. Each study site was asked to submit only clinically significant isolates, as defined by local site criteria, from out- and in-patients with urinary infections. Fifty (2009) to 100 (2007, 2008) consecutive urinary tract isolates per year per medical center site (one isolate per patient) were collected. Primary isolate identification was performed by the submitting medical center site and confirmed by the coordinating laboratory, as required, based on morphological characteristics and spot tests [43]. If an isolate identification made by the coordinating laboratory did not match that provided by the submitting site, the isolate was removed from the study. All isolates were stored at −70 °C in skim milk. 

At the Department of Epidemiology, Biostatistics and Occupational Health of McGill University, the isolates from the McGill University student health service and the community health center, also in Montréal, as well as those from the CANWARD study were recovered on MacConkey and CLED agar (Uricult dipslides: Orion Diagnostica, Oy, Finland). A single representative colony was picked from the MacConkey side of the Uricult slide and grown in LB broth and archived in 15% glycerol at −80 °C. When needed, further testing, based upon indole production and the presence of lysine or ornithine decarboxylase, was also performed to confirm the bacterial identification. 

### 3.2. Culture Conditions

For all experiments, bacteria were grown in LB medium or synthetic urine medium. The latter medium was based on that devised by [44], and its composition and method of sterilization have been described previously [45]. Briefly the medium was composed of CaCl_2_, 0.65 g/L; MgCl_2_, 0.65 g/L; NaCl, 4.6 g/L; Na_2_SO_4_, 2.3 g/L; Na_3_-citrate, 0.65 g/L; Na_2_-oxalate, 0.02 g/L; KH_2_PO_4_, 2.8 g/L; KCl, 1.6 g/L; NH_4_Cl, 1.0 g/L; urea, 25 g/L; creatinine, 1.1 g/L; Luria-Bertani (LB) broth (Difco, Sparks, MD, USA), 10 g/L. The pH of the medium is adjusted to pH 5.8 prior to sterilization by filtration through a 0.2-µm filter.

### 3.3. *E. coli* Serotyping

Identification of somatic (O) and flagellar (H) antigens was performed by standard agglutination methods [46]. The isolates were serotyped at the Public Health Agency of Canada, Laboratory for Foodborne Zoonoses, Guelph, ON, Canada.

### 3.4. Susceptibility Testing

The broth microdilution method was used (Sensititre System; Trek Diagnostics, Cleveland, OH), following the protocols of the Canadian Integrated Program for Antimicrobial Resistance Surveillance [47,48]. Each *E. coli *isolate was tested for the following antimicrobial agents (breakpoints are indicated in parentheses): amikacin (≥64 μg/mL), amoxicillin-clavulanic acid (≥32 and ≥16 µg/mL, respectively), ampicillin (≥32 µg/mL), cefoxitin (≥32 µg/mL), ceftiofur (≥8 μg/mL), ceftriaxone (≥2 µg/mL) (11), chloramphenicol (≥32 µg/mL), ciprofloxacin (≥4 µg/mL), gentamicin (≥16 μg/mL), kanamycin (≥64 μg/mL), naladixic acid (≥32 μg/mL), streptomycin (≥64 μg/mL), sulfisoxazole (≥512 μg/mL), tetracycline (≥16 μg/mL), and trimethoprim-sulfamethoxazole (≥4 and ≥76 μg/mL, respectively).

### 3.5. Bacteriophage Isolation

Three liters of preliminary treated sewage sample from the Guelph water treatment plant were centrifuged at 3,000 RPM for 20 min to eliminate solid debris. The clarified supernatant was concentrated to 400 mL using the tangential flow system as described in [49]. NaCl (20.5 g) was then added to the concentrate and stirred until it dissolved before 40 g of polyethylene glycol was added to precipitate the phage present in the concentrate. This was left overnight while being stirred gently with a magnetic stirrer at 4 °C. Precipitated phage was recovered by centrifugation at 3,000 RPM for 10 min and resuspended in 3 mL SM buffer [50]. This was subjected to two rounds of CsCl gradient purification steps and the resulting viral band isolated.

*E. coli *isolates Can 91, Can 40 and MSHS1210 were used as propagating strains for the isolation of single plaques from serially diluted CsCl purified phage concentrate. All three isolates were from midstream urine specimens with Can 40 and Can 91 being part of the CANWARD study whereas MSHS1210 was from the McGill University student health service. 

LB agar (Difco) plugs from standard agar overlay method [51] were resuspended in 500 µL SM buffer, diluted and used for further single plaque isolation on the respective *E. coli *propagation strains. This was repeated three times for each strain to ensure unique phages for each UPEC isolate. The single unique phages isolated from *E. coli *isolates Can 91, Can 40 and MSHS1210 were named vB_EcoP_ACG-C91, vB_EcoM_ACG-C40 and vB_EcoS_ACG-M12, respectively, following the naming convention of [52].

### 3.6. Transmission Electron Microscopy

Phages were pelleted at 25,000 × g for 1 hour, using a Beckman high-speed centrifuge and a JA-18.1 fixed-angle rotor (Beckman, Palo Alto, CA, USA). Phage pellets were washed twice under the same conditions in neutral 0.1 M ammonium acetate and resuspended. The phages were then deposited on copper grids with carbon-coated Formvar films and stained with 2% uranyl acetate (pH 4) or 2% phosphotungstate (pH 7.2). They were examined in a Philips EM electron microscope. Magnification was calibrated using T4 phage tails as size standards.

### 3.7. Bacteriophage DNA Isolation, Restriction Analysis and Sequencing

To separate phage from bacterial debris, crude phage lysates were centrifuged at 10, 000 × g for 15 min at 4 °C. Bacterial nucleic acids in the supernatants were digested with pancreatic DNase 1 and RNase A, each to a final concentration of 10 μg/mL (Sigma-Aldrich Canada Ltd., Oakville, ON, USA). Phage particles were then precipitated in the presence of 10% w/v (final concentration) polyethylene glycol (PEG-8000; Sigma-Aldrich) at 4 °C overnight. The precipitated phage particles were recovered by centrifugation, resuspended in TM buffer (10 mM Tris-HCl, pH 7.8, 1 mM MgSO_4_). The DNA was extracted from a portion of the precipitated viral particles using the SDS/proteinase K method modified from [50], followed by extraction with phenol:chloroform:isoamyl alcohol (25:24:1, vol/vol), ethanol precipitation and resolution in 10 mM Tris-HCl (pH 7.5). The DNA was characterized spectrophotometrically.

Phage DNA was digested with restriction enzymes BamHI, BglI, EcoRV, HindIII, NaeI, XbaI, NdeI, PstI, and SmaI (New England Biolabs, Hertfordshire, UK), according to the manufacturer's instructions. Phage Lambda DNA (Fermentas Inc., Hanover, MD, USA) digested by the same enzymes was used as a positive control for the restriction digest analysis.

The DNA was subjected to pyrosequencing (454 technology) at the McGill University and Génome Québec Innovation Centre (Montréal, QC, Canada) to between 195 and 930 fold coverage.

### 3.8. Bioinformatic Analysis

The sequences were rearranged to resemble their GenBank homologs and annotated using MyRAST [53]. The generated gbk file was incorporated into Kodon (Applied Maths, Austin, TX, USA) and proof-read. Proteins were generated using gbk2faa program [54] and screened for homologs using BLAST algorithm [55]. In addition they were searched for conserved motifs in the Pfam database [56] and transmembrane helix predictions using TMHMM [57] and Phobius [58]. The sequence was also screened for tRNAs using tRNAscan-SE program [59]. The final sequences were converted to Sequin format [60] using gbk2sqn [61] before deposition into GenBank. The accession numbers are ACG-C91 JN986844, ACG-M12 JN986845 and ACG-C40 JN986846.

### 3.9. Biofilm Assay

Each of the 253 *E. coli *isolates was screened for biofilm formation using the 96-well plate assay as described in [62], with minor modifications. Briefly, *E. coli *isolates were used to inoculate 5 mL of synthetic urine medium and grown for 16 h at 37 °C. A 1:100 dilution of each of the cultures was made in synthetic urine media and 200 µL of each diluted culture was added to six wells in an untreated 96-well polystyrene flat-bottomed, 96-well microtiter plates (Costar; Corning Inc., Corning, NY, USA). The plate was covered and incubated 37 °C for 48 h without agitation. Negative control wells that contained 200 µL of sterile synthetic urine media only were included. Following incubation, planktonic bacteria were removed by pipetting out the culture and washing the wells twice with PBS to remove loosely bound cells, using a multichannel pipettor. A 1% (wt/vol; 210 µL) crystal violet (CV) solution was added to the wells for 10 min. The CV stain was then decanted and the unbound stain removed by washing the plate in a water tray. The plates were left to dry following which 200 µL of 95% ethanol added in each well to solubilize the bound CV from the stained *E. coli *biofilms. The absorbance of CV at 600 nm was measured in a Wallac-Victor^2^ 1420 Multilabel Counter (Perkin-Elmer, Boston, MA, USA).

### 3.10. Bacteriophage Host Range on Biofilm Forming UPEC Isolates

The host range of phages ACG-C91, ACG-C40 and ACG-M12 against biofilm forming *E. coli *UTI isolates was determined by standard spot tests [63]. Briefly, this involved mixing 200 µL overnight culture of each isolate with 3 mL molten top LB agar (0.6% agar) and pouring on 96 mm diameter plates containing bottom agar. This was left to solidify then 10 μl of phage to be tested was spotted in the middle of the plate and left to dry before incubation inverted overnight at 37 °C.

### 3.11. Bacteriophage Activity against Established Biofilms

*E. coli *isolate CLSC 94 was used to test the effect of the two environmentally isolated phages ACG-C40 and ACG-C91 on established biofilms. The method of [64] with minor modifications was used. Briefly, stationery-phase culture of *E. coli *CLSC94 was diluted 1:100 and 200 µL of the inoculum added to 12 wells of an untreated 96-well polystyrene microtiter plate. This was incubated for 24 h at 37 °C without agitation. Negative control wells that contained 200 µL of sterile synthetic urine media only were included. Following incubation period, planktonic bacteria were removed by pipetting out the culture, and washing the wells twice with PBS to remove loosely bound cells. The wells were then treated with 200 µL of ACG-C40 and ACG-C91 phage lysate at titers of 10^5^, 10^7^ and 10^9^ PFU/mL added to each of the 12 wells. Phages were also added to the negative control wells which contained only UPEC cells grown in sterile synthetic urine media. After incubation periods of 2 h, 4 h, 8 h and 24 h, planktonic bacteria were removed and wells were washed twice with PBS to remove loosely bound cells. The remaining biofilm was stained as described above. 

## 4. Conclusions

We were able to isolate phages that are effective against UPECstrains and are able to degrade biofilms formed by the UPEC strains. The phages’ DNA sequences were determined. They were screened and found to be devoid of undesirable laterally transferable virulence and antimicrobial resistance determinants on the basis of homologies with known virulence and resistant genes available in GenBank.

The study resulted in detailed characterization of a large set of UPEC with regards to biofilm forming capabilities and antimicrobial resistance. The candidate phages with adequate lytic spectrum for therapeutic purposes (including efficacy for disruption of existing biofilms) were characterized in detail. The phages have a promising potential for phage therapy of UTIs caused by biofilm forming UPEC.

## Supplementary Files

Supplementary File 1: PDF-Document (PDF, 660 KB)
