# Supplementary material for: Bacteriophages with the Ability to Degrade Uropathogenic Escherichia Coli Biofilms
Source: Viruses. 2012 Apr 10;4(4):471–87. doi: 10.3390/v4040471 (PMC3347319; doi:10.3390/v4040471)
Supplement: Supplementary File 1: — PDF-Document (PDF, 660 KB) [file viruses-04-00471-s001.pdf]

Figure S1. Genetic and physical map of phage ACG-C40 (adapted from the CGView output).

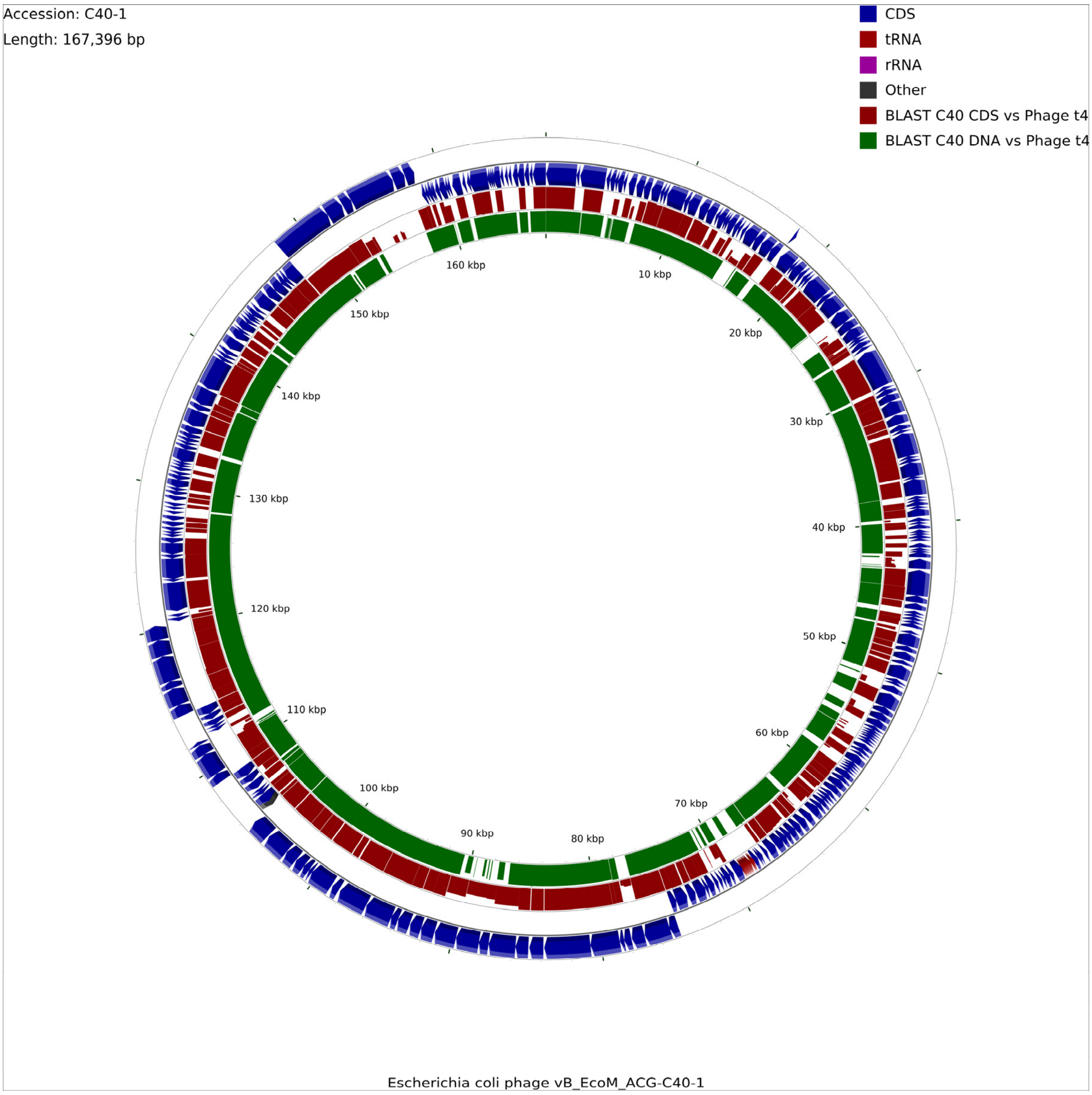

Figure S2. Alignment of: (**a**) ACG-C91 genome and phage SP6 genome; (**b**) ACG-M12 genome and phage RTP genome with Mauve v.2.3.0.

(**a**)

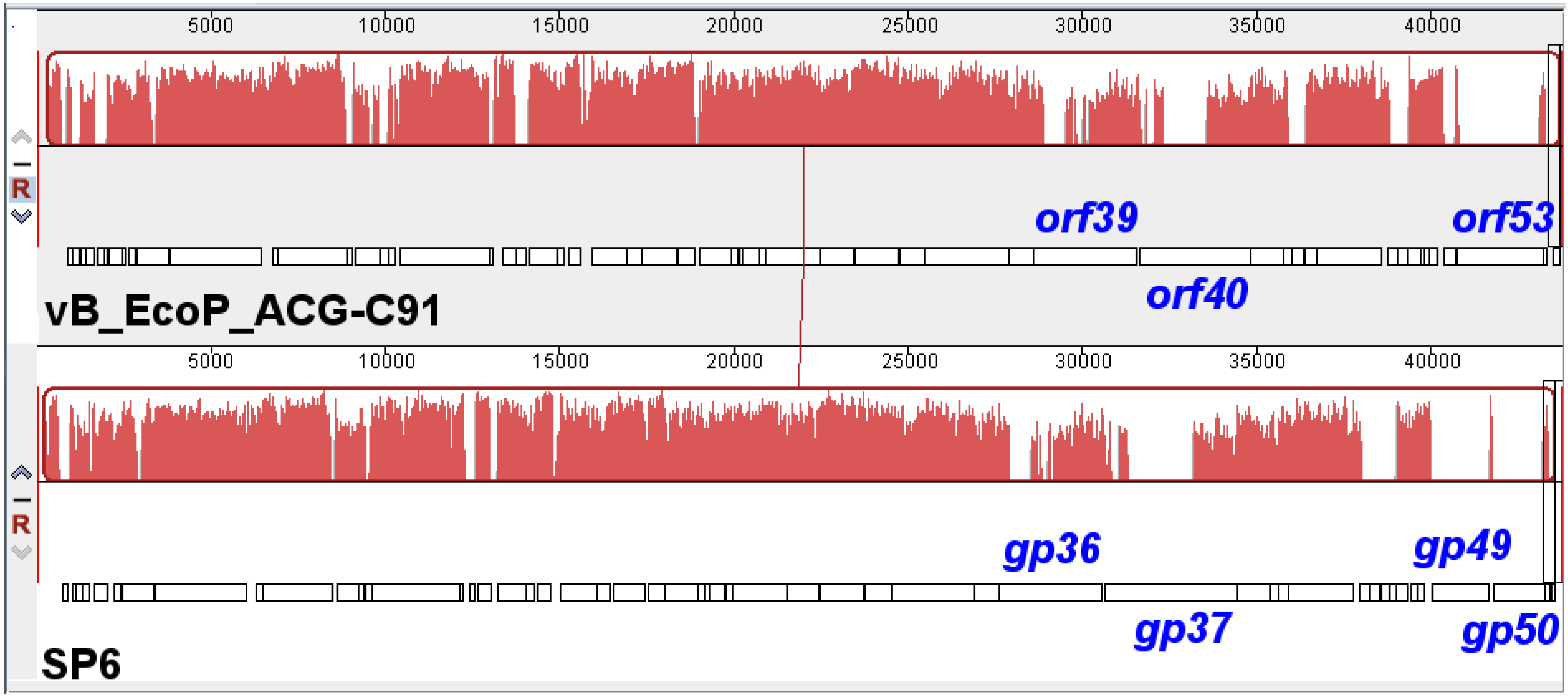

Figure S2. *Cont.*

(**b**)

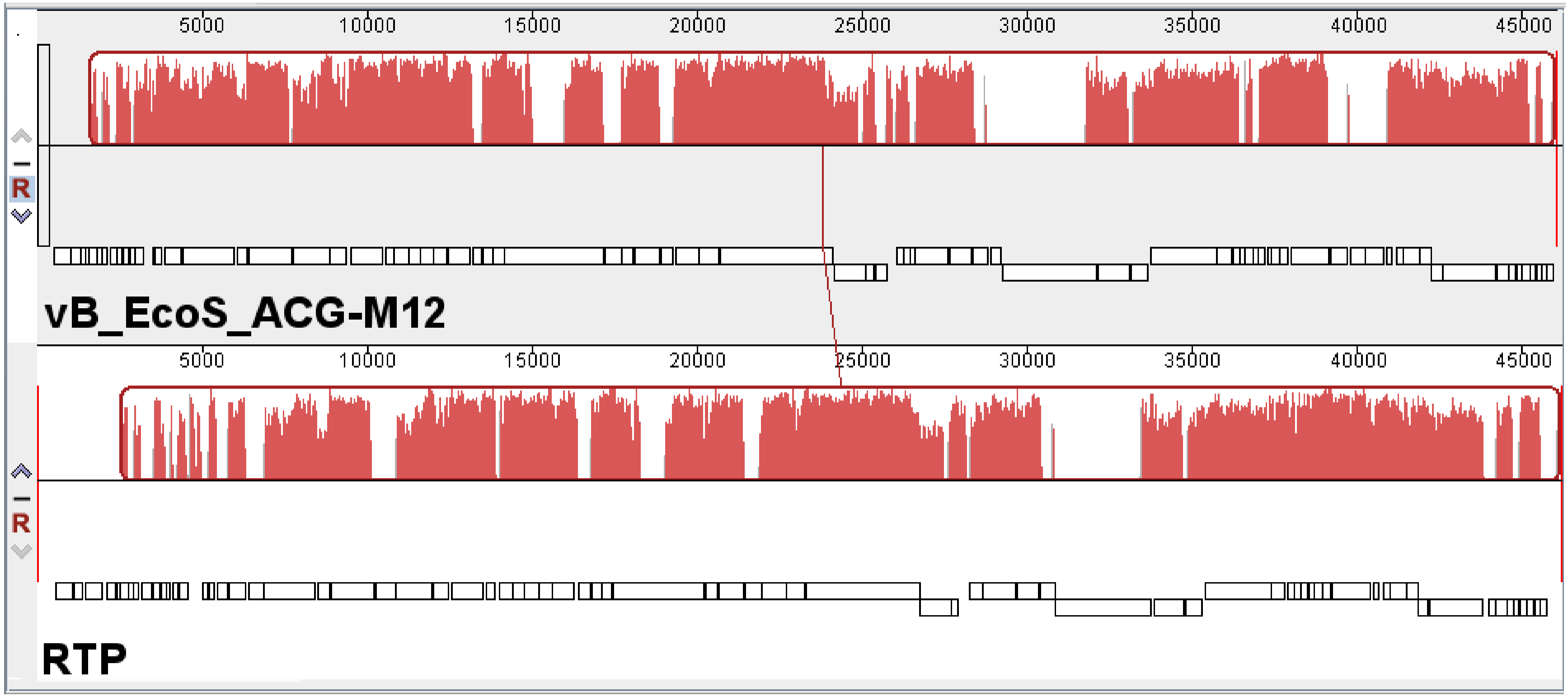

Table S1. Summary of ORFs and proteins encoded by phage ACG-C40.

Coordinates	Strand	Length	Gene	Product
1..2178	-	2178	orf001	protector from prophage-induced early lysis rIIA
2189..2392	-	204	orf002	conserved hypothetical protein; Enterobacteria phage T4 rIIA.1 homolog
2447..4264	-	1818	orf003	topoisomerase II, large subunit, N-terminal region; Enterobacteriophage RB14 DNA topoisomerase subunit gp60plus39 homolog
4334..4594	-	261	orf004	conserved hypothetical protein; Enterobacteria phage T4 gp39.1 homolog
4600..4971	-	372	orf005	conserved hypothetical protein
4974..5150	-	177	orf006	conserved hypothetical protein; Enterobacteria phage T4 gp39.2 homolog
5153..5566	-	414	orf007	mRNA metabolism modulator goF
5566..5781	-	216	orf008	modifier of suppressor tRNAs cef
5954..6445	-	492	orf009	modifier of transcription MotB
6519..7067	-	549	orf010	conserved hypothetical protein; Enterobacteria phage T4 hypothetical protein MotB.1 homolog
7070..7570	-	501	orf011	conserved hypothetical protein; Enterobacteria phage T4 hypothetical protein MotB.2 homolog
7634..8317	-	684	orf012	exonuclease A DexA
8317..8559	-	243	orf013	conserved hypothetical protein; Enterobacteria phage T4 hypothetical protein DexA.1 homolog
8552..8797	-	246	orf014	conserved hypothetical protein; Enterobacteria phage T4 hypothetical protein DexA.2 homolog
8819..10138	-	1320	orf015	DNA helicase Dda
10135..10446	-	312	orf016	conserved hypothetical protein; Enterobacteria phage T4 hypothetical protein Dda.1 homolog
10448..11194	-	747	orf017	anti-sigma factor Srd
11318..11920	-	603	orf018	RNA polymerase ADP-ribosylase ModA
11917..12540	-	624	orf019	ADP-ribosylase ModB
12608..12790	-	183	orf020	conserved hypothetical protein; Enterobacteria phage T4 hypothetical protein ModA.2 homolog
12799..13269	-	471	orf021	conserved hypothetical protein; Enterobacteria phage T4 hypothetical protein ModA.3 homolog
13262..13426	-	165	orf022	conserved hypothetical protein; Enterobacteria phage T4 hypothetical protein ModA.4 homolog
13423..13578	-	156	orf023	transcription modulator Srh
13601..14086	-	486	orf024	transcription modulator under heat shock Mrh
14095..14433	-	339	orf025	conserved hypothetical protein; Enterobacteria phage T4 hypothetical protein Mrh.1 homolog
14433..14639	-	207	orf026	conserved hypothetical protein; Enterobacteria phage T4 hypothetical protein Mrh.2 homolog
14738..14974	-	237	orf027	small outer capsid protein Soc

Table S1. *Cont.*

Coordinates	Strand	Length	Gene	Product
15009..15845	-	837	orf028	conserved hypothetical protein; Enterobacteria phage T4 homing endonuclease SegA homolog
15835..16353	-	519	orf029	dCTPase
16414..17193	-	780	orf030	DNA adenine methyltransferase Dam
17193..17753	-	561	orf031	hypothetical protein
17853..18053	+	201	orf032	conserved hypothetical protein
18050..19078	-	1029	orf033	DNA primase subunit
19082..19246	-	165	orf034	conserved hypothetical protein; Enterobacteria phage T4 orf 61.1 homolog
19248..19874	-	627	orf035	conserved hypothetical protein; Enterobacteria phage T4 orf 61.2 homolog
19874..20167	-	294	orf036	spackle periplasmic protein sp
20228..20485	-	258	orf037	conserved hypothetical protein; Enterobacteria phage T4 orf 61.4 homolog
20487..20669	-	183	orf038	discriminator of mRNA degradation Dmd
20728..22155	-	1428	orf039	DNA primase-helicase subunit
22165..22509	-	345	orf040	head vertex assembly chaperone
22502..23683	-	1182	orf041	RecA-like recombination protein UvsX
23761..24603	-	843	orf042	conserved hypothetical protein
24600..25241	-	642	orf043	conserved hypothetical protein; Enterobacteria phage T4 endodeoxyribonuclease segC homolog
25232..25972	-	741	orf044	dCMP hydroxymethylase
25980..26129	-	150	orf045a	conserved hypothetical protein
26126..26377	-	252	orf045	immunity to superinfection membrane protein Imm
26385..26765	-	381	orf046	conserved hypothetical protein; Enterobacteria phage T4 hypothetical predicted membrane protein Imm.1 homolog
26776..27012	-	237	orf047	conserved hypothetical protein
27196..29892	-	2697	orf048	DNA polymerase; Enterobacteria phage T4 gp43 homolog
29971..30192	-	222	orf049	conserved hypothetical protein
30194..30562	-	369	orf050	translational repressor protein RegA
30564..31127	-	564	orf051	clamp loader subunit, DNA polymerase accessory protein; Enterobacteria phage T4 gp62 homolog
31129..32088	-	960	orf052	clamp loader subunit, DNA polymerase accessory protein; Enterobacteria phage T4 gp44 homolog
32140..32826	-	687	orf053	sliding clamp, DNA polymerase accessory protein; Enterobacteria phage T4 gp45 homolog

Table S1. *Cont.*

Coordinates	Strand	Length	Gene	Product
32882..33271	-	390	orf054	RNA polymerase binding protein RpbA
33281..33469	-	189	orf055	conserved hypothetical protein; Enterobacteria phage T4 conserved hypothetical protein gp45.2 homolog
33525..35207	-	1683	orf056	recombination endonuclease subunit; Enterobacteria phage T4 gp46 homolog
35204..35410	-	207	orf057	conserved hypothetical protein; Enterobacteria phage T4 hypothetical protein gp46.1 homolog
35391..35654	-	264	orf058	conserved hypothetical protein; Enterobacteria phage T4 hypothetical protein gp46.2 homolog
35651..36670	-	1020	orf059	recombination endonuclease subunit; Enterobacteria phage T4 gp47 homolog
36667..36807	-	141	orf60a	conserved hypothetical protein; Enterobacteria phage T4 hypothetical predicted membrane protein gp47.1 homolog
36847..38049	-	1203	orf060	alpha glucosyl transferase a-gt
38116..38289	-	174	orf061	conserved hypothetical protein; Enterobacteria phage T4 orfC homolog
38293..38496	-	204	orf062	conserved hypothetical protein; Enterobacteria phage T4 hypothetical protein a-gt.3 homolog
38465..38782	-	318	orf063	conserved hypothetical protein; Enterobacteria phage T4 hypothetical protein a-gt.4 homolog
38784..38972	-	189	orf064	conserved hypothetical protein; Enterobacteria phage T4 hypothetical protein a-gt.5 homolog
38986..39543	-	558	orf065	sigma factor recognition late T4 promoters; Enterobacteria phage T4 gp55 homolog
39622..39891	-	270	orf066	conserved hypothetical protein
39888..40103	-	216	orf067	conserved hypothetical protein; Enterobacteria phage T4 hypothetical protein gp55.1 homolog
40106..40432	-	327	orf068	conserved hypothetical protein; Enterobacteria phage T4 hypothetical protein gp55.2 homolog
40485..40685	-	201	orf069	conserved hypothetical protein; Enterobacteria phage T4 hypothetical protein gp55.3 homolog
40686..40817	-	132	orf070	conserved hypothetical protein; Enterobacteria phage T4 conserved hypothetical protein gp55.4 homolog
40825..41118	-	294	orf071	conserved hypothetical protein; Enterobacteria phage T4 conserved hypothetical protein gp55.5 homolog
41111..41293	-	183	orf072	conserved hypothetical protein; Enterobacteria phage T4 conserved hypothetical protein gp55.6 homolog
41452..41760	-	309	orf073	Glutaredoxin NrdH
41763..41975	-	213	orf074	conserved hypothetical protein; Enterobacteria phage T4 conserved hypothetical predicted membrane protein gp55.8 homolog
41985..42098	-	114	orf075	conserved hypothetical protein
42091..42483	-	393	orf076	anaerobic NTP reductase, small subunit, NrdG
42558..43292	-	735	orf077	homing endonuclease SegD
43381..45198	-	1818	orf078	anaerobic NTP reductase, large subunit, NrdD

Table S1. *Cont.*

Coordinates	Strand	Length	Gene	Product
45195..45668	-	474	orf079	packaging and recombination endonuclease VII, EndoVII
45710..46195	-	486	orf080	protease inhibitor Pin
46286..46513	-	228	orf081	conserved hypothetical protein
46492..46707	-	216	orf082	conserved hypothetical protein
46704..46967	-	264	orf083	thioredoxin NrdC
46969..47211	-	243	orf084	conserved hypothetical protein; Enterobacteria phage T4 conserved hypothetical protein NrdC.1 homolog
47198..47512	-	315	orf085	conserved hypothetical protein; Enterobacteria phage T4 conserved hypothetical protein NrdC.2 homolog
47708..47923	-	216	orf086	conserved hypothetical protein; Enterobacteria phage T4 conserved hypothetical protein NrdC.5 homolog
47932..48819	-	888	orf087	conserved hypothetical protein; Enterobacteria phage T4 conserved hypothetical protein NrdC.6 homolog
48831..49235	-	405	orf088	conserved hypothetical protein; Enterobacteria phage T4 conserved hypothetical NrdC.7 homolog
49291..49818	-	528	orf089	conserved hypothetical protein; Enterobacteria phage T4 conserved hypothetical protein NrdC.8 homolog
49878..50180	-	303	orf090	conserved hypothetical protein; Enterobacteria phage T4 conserved hypothetical protein NrdC.9 homolog
50283..51251	-	969	orf091	conserved hypothetical protein; Enterobacteria phage T4 conserved hypothetical protein NrdC.10 homolog
51321..51584	-	264	orf092	conserved hypothetical protein
51581..51730	-	150	orf093	conserved hypothetical protein
51855..52340	-	486	orf094	ADP-ribosylase ModB
52468..53478	-	1011	orf095	conserved hypothetical protein; Enterobacteria phage T4 conserved hypothetical protein NrdC.11 homolog
53478..53939	-	462	orf096	conserved hypothetical protein
53942..54463	-	522	orf097	conserved hypothetical protein
54470..55003	-	534	orf098	conserved hypothetical protein; Enterobacteria phage T4 conserved hypothetical protein MobD.1 homolog
55005..55271	-	267	orf099	conserved hypothetical protein
55273..55458	-	186	orf100	conserved hypothetical protein
55458..55622	-	165	orf101a	conserved hypothetical protein; Enterobacteria phage T4 conserved hypothetical protein MobD.2
55623..55796	-	174	orf101	conserved hypothetical protein; Enterobacteria phage T4 hypothetical protein MobD.2a homolog
55786..55980	-	195	orf102	conserved hypothetical protein; Enterobacteria phage T4 hypothetical protein MobD.3 homolog
55983..56186	-	204	orf103	conserved hypothetical protein; Enterobacteria phage T4 hypothetical protein MobD.4
56186..56374	-	189	orf104	conserved hypothetical protein; Enterobacteria phage T4 hypothetical protein MobD.5 homolog
56470..56856	-	387	orf105	conserved hypothetical protein; Enterobacteria phage T4 hypothetical protein MobD.6 homolog
56853..57146	-	294	orf106	lysis inhibition regulator, membrane protein rI

Table S1. *Cont.*

Coordinates	Strand	Length	Gene	Product
57159..57371	-	213	orf107	conserved hypothetical protein; Enterobacteria phage T4 conserved hypothetical protein rI.1 homolog
57414..57995	-	582	orf108	thymidine kinase
58005..58190	-	186	orf109	conserved hypothetical protein; Enterobacteria phage T4 conserved hypothetical protein Tk.2 homolog
58187..58360	-	174	orf110	conserved hypothetical protein
58357..58563	-	207	orf111	conserved hypothetical protein
58560..58772	-	213	orf112	conserved hypothetical protein; Enterobacteria phage T4 conserved hypothetical proteinTk.2 homolog
58769..59236	-	468	orf113	conserved hypothetical protein; Enterobacteria phage T4 conserved hypothetical protein Tk.4 homolog
59233..59574	-	342	orf114	valyl-tRNA synthetase modifier Vs
59578..60111	-	534	orf115	conserved hypothetical protein; Enterobacteria phage T4 conserved hypothetical protein Vs.1 homolog
60119..60580	-	462	orf116	site-specific RNA endonuclease RegB
60640..60918	-	279	orf117	conserved hypothetical protein; Enterobacteria phage T4 conserved hypothetical protein Vs.3 homolog
60918..61184	-	267	orf118	conserved hypothetical protein; Enterobacteria phage T4 conserved hypothetical protein Vs.4 homolog
61177..61398	-	222	orf119	conserved hypothetical protein
61398..61760	-	363	orf120	conserved hypothetical protein; Enterobacteria phage T4 conserved hypothetical protein Vs.6 homolog
61767..62096	-	330	orf121	conserved hypothetical protein; Enterobacteria phage T4 conserved hypothetical protein Vs.7 homolog
62093..62635	-	543	orf122	conserved hypothetical protein; Enterobacteria phage T4 conserved hypothetical protein Vs.8 homolog
62777..63250	-	474	orf123	conserved hypothetical protein;
63339..63833	-	495	orf124	lysozyme murein hydrolase e
63871..64311	-	441	orf125	nudix hydrolase
64308..64796	-	489	orf126	conserved hypothetical protein; Enterobacteria phage T4 conserved hypothetical, predicted membrane protein e.2 homolog
64793..65167	-	375	orf127	conserved hypothetical protein; Enterobacteria phage T4 conserved hypothetical predicted membrane protein e.3 homolog
65149..65541	-	393	orf128	conserved hypothetical protein; Enterobacteria phage T4 conserved hypothetical, predicted membrane protein e.4 homolog
65510..66118	-	609	orf129	conserved hypothetical protein; Enterobacteria phage T4 conserved hypothetical proteine.5 homolog
66166..66759	-	594	orf130	conserved hypothetical protein; Enterobacteria phage T4 conserved hypothetical protein e.6 homolog
66864..67127	-	264	orf131	conserved hypothetical protein; Enterobacteria phage T4 conserved hypothetical protein e.8 homolog
67325..67465	-	141	orf132b	hypothetical protein

Table S1. *Cont.*

Coordinates	Strand	Length	Gene	Product
67469..67612	-	144	orf132a	conserved hypothetical protein
67674..68024	-	351	orf132	conserved hypothetical protein
68996..69604	-	609	orf133	conserved hypothetical protein; Enterobacteria phage T5 H-N-H endonuclease TflIV homolog
69715..70002	-	288	orf134	conserved hypothetical protein; Enterobacteria phage T4 conserved hypothetical protein Trna.2 homolog
70005..70403	-	399	orf135	conserved hypothetical protein; Enterobacteria phage T4 conserved hypothetical protein Trna.3 homolog
70403..70588	-	186	orf136	conserved hypothetical protein; Enterobacteria phage T4 conserved hypothetical predicted membrane protein Trna.4 homolog
70647..70880	-	234	orf137	conserved hypothetical protein; Enterobacteria phage T6 putative internal head protein Ip6 homolog
70957..71214	-	258	orf138	conserved hypothetical protein; Enterobacteria phage T6 hypothetical protein Ip5 homolog
71287..71742	-	456	orf139	conserved hypothetical protein; Enterobacteria phage T4 conserved hypothetical protein gp57B homolog
71742..71984	-	243	orf140	chaperone for tail fiber formation; Enterobacteria phage T4 gp57A homolog
71984..72709	-	726	orf141	dNMP kinase; Enterobacteria phage T4 gp1 homolog
72759..73289	-	531	orf142	tail completion and sheath stabilizer protein; Enterobacteria phage T4 gp3 homolog
73396..74220	-	825	orf143	DNA end protector protein; Enterobacteria phage T4 gp2 homolog
74220..74672	-	453	orf144	head completion protein; Enterobacteria phage T4 gp4 homolog
74720..75310	+	591	orf145	baseplate wedge subunit; Enterobacteria phage T4 gp53 homolog
75294..77024	+	1731	orf146	baseplate hub subunit and tail lysozyme; Enterobacteria phage T4 gp5 homolog
77044..77910	+	867	orf147	conserved hypothetical protein; Enterobacteria phage T4 SegA homing endonuclease homolog
77945..78439	+	495	orf148	conserved hypothetical protein; Enterobacteria phage T4 conserved hypothetical protein gp5.1 homolog
78440..78733	+	294	orf149	conserved hypothetical protein; Enterobacteria phage T4 conserved hypothetical protein gp5.4 homolog
78742..80724	+	1983	orf150	baseplate wedge subunit; Enterobacteria phage T4 gp6 homolog
80721..83819	+	3099	orf151	baseplate wedge initiator; Enterobacteria phage T4 gp7 homolog
83872..84816	+	945	orf152	baseplate wedge subunit; Enterobacteria phage T4 gp8 homolog
84880..85746	+	867	orf153	baseplate wedge tail fiber connector; Enterobacteria phage T4 gp9 homolog
85746..87551	+	1806	orf154	baseplate wedge subunit and tail pin; Enterobacteria phage T4 gp10 homolog
87551..88210	+	660	orf155	baseplate wedge completion tail pin; Enterobacteria phage T4 gp11 homolog
88207..89757	+	1551	orf156	short tail fibers; Enterobacteria phage T4 gp12 homolog
89767..91224	+	1458	orf157	fibritin neck whiskers; Enterobacteria phage T4 fibritin neck whiskers Wac homolog
91256..92185	+	930	orf158	neck protein; Enterobacteria phage T4 gp13 homolog

Table S1. *Cont.*

Coordinates	Strand	Length	Gene	Product
92187..92957	+	771	orf159	neck protein; Enterobacteria phage T4 gp14 homolog
92999..93817	+	819	orf160	tail sheath stabilizer and completion protein; Enterobacteria phage T4 gp15 homolog
93826..94320	+	495	orf161	terminase DNA packaging enzyme, small subunit; Enterobacteria phage T4 gp16 homolog
94304..96136	+	1833	orf162	terminase DNA packaging enzyme, large subunit; Enterobacteria phage T4 gp17 homolog
96168..98147	+	1980	orf163	tail sheath monomer; Enterobacteria phage T4 gp18 homolog
98264..98755	+	492	orf164	tail tube protein; Enterobacteria phage T4 gp19 homolog
98839..100413	+	1575	orf165	portal vertex protein of head; Enterobacteria phage T4 gp20 homolog
100413..100652	+	240	orf166	prohead core protein, precursor to internal peptides; Enterobacteria phage T4 gp67 homolog
100652..101077	+	426	orf167	prohead core protein; Enterobacteria phage T4 gp68 homolog
101077..101715	+	639	orf168	prohead core scaffold protein and protease; Enterobacteria phage T4 gp21 homolog
101746..102555	+	810	orf169	prohead core scaffold protein; Enterobacteria phage T4 gp22 homolog
102574..104133	+	1560	orf170	major capsid protein; Enterobacteria phage T4 gp23 homolog
104217..105500	+	1284	orf171	head vertex protein; Enterobacteria phage T4 gp24 homolog
105531..105773	-	243	orf172	RNA ligase 2 RnlB
105891..106535	-	645	orf173	RNA ligase 2 RnlB
106545..106823	-	279	orf174	conserved hypothetical protein; Enterobacteria phage T4 conserved hypothetical protein gp24.2 homolog
106810..106995	-	186	orf175	conserved hypothetical protein; Enterobacteria phage T4 conserved hypothetical protein gp24.3 homolog
107127..107687	-	561	orf176	head outer capsid protein Hoc
107749..108258	-	510	orf177	head outer capsid protein Hoc
108268..108948	-	681	orf178	inhibitor of prohead protease Inh
108998..109615	+	618	orf179	homing endonuclease SegA
109618..111126	+	1509	orf180	RNA-DNA and DNA-DNA helicase, ATPase UvsW
111123..111815	+	693	orf181	homing endonuclease MobE
111841..112071	+	231	orf182	RNA-DNA and DNA-DNA helicase, ATPase UvsW
112127..112294	-	168	orf183	conserved hypothetical protein; Enterobacteria phage T4 conserved hypothetical protein UvsY.-2 homolog
112323..112547	-	225	orf184	conserved hypothetical protein; Enterobacteria phage T4 conserved hypothetical protein UvsY.-1 homolog
112547..112960	-	414	orf185	recombination, repair and ssDNA binding protein UvsY
113027..113425	-	399	orf186	baseplate wedge subunit; Enterobacteria phage T4 gp25 homolog
113425..114051	-	627	orf187	baseplate hub; Enterobacteria phage T4 gp26 homolog

Table S1. *Cont.*

Coordinates	Strand	Length	Gene	Product
114102..114854	+	753	orf188	baseplate hub assembly protein; Enterobacteria phage T4 gp51 homolog
114851..116023	+	1173	orf189	baseplate hub subunit; Enterobacteria phage T4 gp27 homolog
116046..116504	+	459	orf190	baseplate hub distal subunit; Enterobacteria phage T4 gp28 homolog
116501..118273	+	1773	orf191	baseplate hub subunit, tail length determinator; Enterobacteria phage T4 gp29 homolog
118282..119376	+	1095	orf192	baseplate tail tube cap; Enterobacteria phage T4 gp48 homolog
119376..120341	+	966	orf193	baseplate tail tube initiator; Enterobacteria phage T4 gp54 homolog
120370..120660	-	291	orf194	conserved hypothetical protein; Enterobacteria phage T4 conserved hypothetical protein Alt.-3 homolog
120690..120896	-	207	orf195	conserved hypothetical protein; Enterobacteria phage T4 hypothetical protein Alt.-2 homolog
121082..123130	-	2049	orf196	RNA polymerase ADP-ribosylase Alt
123183..123371	-	189	orf197	conserved hypothetical protein; Enterobacteria phage T4 conserved hypothetical protein Alt.1 homolog
123368..124831	-	1464	orf198	DNA ligase; Enterobacteria phage T4 gp30 homolog
124828..125097	-	270	orf199	conserved hypothetical protein; Enterobacteria phage T4 conserved hypothetical protein gp30.1 homolog
125097..125933	-	837	orf200	conserved hypothetical protein; Enterobacteria phage T4 conserved hypothetical protein gp30.2 homolog
125930..126310	-	381	orf201	conserved hypothetical protein; Enterobacteria phage T4 conserved hypothetical protein gp30.3 homolog
126381..126587	-	207	orf202	conserved hypothetical protein; Enterobacteria phage T4 conserved hypothetical protein gp30.4 homolog
126584..126781	-	198	orf203	conserved hypothetical protein; Enterobacteria phage T4 hypothetical proteingp30.5 homolog
126781..127068	-	288	orf204	conserved hypothetical protein; Enterobacteria phage T4 conserved hypothetical protein gp30.6 homolog
127110..127475	-	366	orf205	conserved hypothetical protein; Enterobacteria phage T4 conserved hypothetical protein gp30.7 homolog
127544..127876	-	333	orf206	conserved hypothetical protein; Enterobacteria phage T4 conserved hypothetical protein gp30.8 homolog
127987..128205	-	219	orf207	conserved hypothetical protein; Enterobacteria phage T4 conserved hypothetical protein gp30.9 homolog
128310..128531	-	222	orf208a	hypothetical protein
128561..128809	-	249	orf208	lysis inhibition accessory protein, rapid lysis phenotype rIII
128957..129292	-	336	orf209	head assembly cochaperone with GroEL
129349..129657	-	309	orf210	conserved hypothetical protein; Enterobacteria phage T4 conserved hypothetical protein gp31.1 homolog
129658..129894	-	237	orf211	conserved hypothetical protein; Enterobacteria phage T4 hypothetical protein gp31.2 homolog
129894..130475	-	582	orf212	dCMP deaminase Cd
130472..130807	-	336	orf213	conserved hypothetical protein; Enterobacteria phage T4 hypothetical protein Cd.1 homolog
130807..131043	-	237	orf214	conserved hypothetical protein; Enterobacteria phage T4 conserved hypothetical protein Cd.2 homolog
131106..131381	-	276	orf215	conserved hypothetical protein; Enterobacteria phage T4 conserved hypothetical protein Cd.3 homolog

Table S1. *Cont.*

Coordinates	Strand	Length	Gene	Product
131384..131584	-	201	orf216	conserved hypothetical protein; Enterobacteria phage T4 conserved hypothetical protein Cd.4 homolog
131577..131774	-	198	orf217	conserved hypothetical protein; Enterobacteria phage T4 hypothetical protein Cd.5 homolog
131774..132682	-	909	orf218	polynucleotide 5'-kinase and 3'-phosphatase PseT
132679..132999	-	321	orf219	conserved hypothetical protein
132996..133226	-	231	orf220	conserved hypothetical protein; Enterobacteria phage T4 conserved hypothetical protein PseT.1 homolog
133223..133522	-	300	orf221	conserved hypothetical protein; Enterobacteria phage T4 conserved hypothetical protein PseT.2 homolog
133519..133872	-	354	orf222	conserved hypothetical protein; Enterobacteria phage T4conserved hypothetical predicted membrane protein PseT.3 homolog
133863..134369	-	507	orf223	inhibitor of host transcription Alc
134432..135556	-	1125	orf224	RNA ligase 1 and tail fiber attachment catalyst RnlA
135609..136019	-	411	orf225	endonuclease II DenA
136047..137225	-	1179	orf226	aerobic NDP reductase, small subunit NrdB
137277..139541	-	2265	orf227	ribonucleoside-diphosphate reductase subunit alpha NrdA
139532..139792	-	261	orf228	conserved hypothetical protein; Enterobacteria phage T4 conserved hypothetical protein NrdA.1 homolog
139812..140075	-	264	orf229	conserved hypothetical protein; Enterobacteria phage T4 conserved hypothetical protein NrdA.2 homolog
140099..140413	-	315	orf230	dTMP thymidylate synthase
140558..141295	-	738	orf231	homing endonuclease I-TevI
141424..141975	-	552	orf232	thymidylate synthase
141975..142103	-	129	orf233	hypothetical protein
142178..142759	-	582	orf234	dihydrofolate reductase Frd
142759..143004	-	246	orf235	conserved hypothetical protein
143015..143257	-	243	orf236	conserved hypothetical protein; Enterobacteria phage T4 conserved hypothetical protein Frd.1 homolog
143268..143336	-	69	orf237a	hypothetical protein
143396..143782	-	387	orf237	conserved hypothetical protein; Enterobacteria phage T4 ORF frd.2 homolog
143828..144055	-	228	orf238	conserved hypothetical protein; Enterobacteria phage T4 hypothetical protein Frd.3 homolog
144202..145107	-	906	orf239	single-stranded DNA binding protein; Enterobacteria phage T4 gp32 homolog
145167..145799	-	633	orf240	homing endonuclease SegG
145801..146454	-	654	orf241	loader of DNA helicase; Enterobacteria phage T4 gp59 homolog
146451..146789	-	339	orf242	late promoter transcription accessory protein; Enterobacteria phage T4 gp33 homolog

Table S1. *Cont.*

Coordinates	Strand	Length	Gene	Product
146767..147036	-	270	orf243	dsDNA binding protein, late transcription DsbA
147045..147962	-	918	orf244	ribonuclease RNaseH
148067..151936	+	3870	orf245	long tail fiber proximal subunit; Enterobacteria phage T4 gp34 homolog
151945..153060	+	1116	orf246	hinge connector of long tail fiber, proximal connector; Enterobacteria phage T4 gp35 homolog
153123..153773	+	651	orf247	hinge connector of long tail fiber, distal connector; Enterobacteria phage T4 gp36 homolog
153782..156874	+	3093	orf248	long tail fiber, distal subunit; Enterobacteria phage T4 gp37 homolog
156911..157711	+	801	orf249	conserved hypothetical protein; Enterobacteria phage RB32 gp38 homolog
157742..158398	+	657	orf250	holin lysis mediator t
158399..158671	-	273	orf251	anti-sigma 70 protein AsiA
158684..158836	-	153	orf252	conserved hypothetical protein; Enterobacteria phage T4 hypothetical protein AsiA.1 homolog
158833..159111	-	279	orf253	inhibitor of MrcBC restriction endonuclease (anti-restriction nuclease) Arn
159101..159220	-	120	orf254	conserved hypothetical protein
159195..159326	-	132	orf255	conserved hypothetical protein; Enterobacteria phage T4 conserved hypothetical protein Arn.1 homolog
159397..159693	-	297	orf256	conserved hypothetical protein; Enterobacteria phage T4 conserved hypothetical protein Arn.2 homolog
159693..160160	-	468	orf257	conserved hypothetical protein; Enterobacteria phage T4 conserved hypothetical protein Arn.3
160157..160312	-	156	orf258	conserved hypothetical protein
160309..160515	-	207	orf259	conserved hypothetical protein; Enterobacteria phage T4 conserved hypothetical protein Arn.4 homolog
160649..161284	-	636	orf260	activator of middle period transcription MotA
161390..161617	-	228	orf261	conserved hypothetical protein
161753..161902	-	150	orf262	hypothetical protein
161899..163227	-	1329	orf263	DNA topoisomerase II medium subunit; Enterobacteria phage T4 gp52 homolog
163232..163372	-	141	orf264a	conserved hypothetical protein; Enterobacteria phage T4 conserved hypothetical predicted membrane protein gp52.1 homolog
163365..163523	-	159	orf264	acridine resistance protein Ac
163531..163611	-	81	orf265a	activator of host PrrC lysyl-tRNA endonuclease Stp
163611..164123	-	513	orf265	nucleoid disruption protein Ndd
164128..164343	-	216	orf266	conserved hypothetical protein; Enterobacteria phage T4 conserved hypothetical protein Ndd.1 homolog
164459..164656	-	198	orf267	conserved hypothetical protein; Enterobacteria phage T4 hypothetical predicted periplasmic protein Ndd.2a homolog

Table S1. *Cont.*

Coordinates	Strand	Length	Gene	Product
164761..164874	-	114	orf268	hypothetical protein
165021..165383	-	363	orf269	conserved hypothetical protein
165365..165841	-	477	orf270	DNA endonuclease IV DenB
165855..166184	-	330	orf271	conserved hypothetical protein; Enterobacteria phage T4 ORF37 homolog
166224..166418	-	195	orf272	conserved hypothetical protein; Enterobacteria phage T4 hypothetical protein DenB.1 homolog
166447..167385	-	939	orf273	protector from prophage-induced early lysis rIIB

Table S2. Summary of ORFs and proteins encoded by phage ACG-C91.

Gene	Coordinates	Strand	Length	Product
orf01	860..997	+	138	conserved hypothetical protein; Enterobacteria phage SP6 gp2 homolog
orf02	994..1221	+	228	hypothetical protein
orf03	1259..1387	+	129	conserved hypothetical protein; Enterobacteria phage K1-5 hypothetical protein EPKV1_gp01 homolog
orf04	1387..1611	+	225	conserved hypothetical protein; Enterobacteria phage K1-5 hypothetical protein 2 homolog
orf05	1741..1908	+	168	hypothetical protein
orf06	1862..1999	+	138	hypothetical protein
orf07	2063..2419	+	357	conserved hypothetical protein; Enterobacteria phage T7 protein 0.3 homolog
orf08	2416..2535	+	120	conseved hypothetical protein; Enterobacteria phage SP6 protein 4 homolog
orf09	2612..2797	+	186	conserved hypothetical protein; Enterobacteria phage K1-5 protein 5 homolog
orf10	2859..3743	+	885	conserved hypothetical protein; Enterobacteria phage SP6 gp7 homolog
orf11	3807..6434	+	2628	putative RNA polymerase
orf12	6760..6888	+	129	conserved hypothetical protein; Enterobacteria phage SP6 gp9 homolog
orf13	6892..8880	+	1989	putative DNA helicase
orf14	8880..9041	+	162	conserved hypothetical protein; Enterobacteria phage K1E hypothetical protein PK1Ep14
orf15	9114..9836	+	723	conserved hypothetical protein; Enterobacteria phage SP6 gp11 homolog
orf16	9847..10065	+	219	conserved hypothetical protein;Enterobacteria phage K1E hypothetical protein PK1Ep16 homolog
orf17	10055..10264	+	210	conserved hypothetical protein; Enterobacteria phage SP6 gp12 homolog
orf18	10416..12962	+	2547	putative DNA polymerase
orf19	12962..13078	+	117	conserved hypothetical protein; Enterobacteria phage K1E hypothetical protein PK1Ep23 homolog
orf20	13332..13709	+	378	conserved hypothetical protein; Enterobacteria phage SP6 gp17 homolog
orf21	13719..14015	+	297	conserved hypothetical protein; Enterobacteria phage K1E hypothetical protein PK1Ep26 homolog
orf22	14093..14899	+	807	conserved hypothetical protein; Enterobacteria phage SP6 gp18 homolog
orf 22A	14900..15127	+	228	conserved hypothetical protein; Enterobacteria phage SP6 gp19 homolog
orf23	15233..15604	+	372	conserved hypothetical protein; Enterobacteria phage SP6 gp20 homolog
orf24	15907..16932	+	1026	exonucluease; Enterobacteria phage SP6 gp21 homolog
orf25	16917..17330	+	414	conserved hypothetical protein; Enterobacteria phage SP6 gp22 homolog

Table S2. *Cont.*

Gene	Coordinates	Strand	Length	Product
orf26	17323..18330	+	1008	conserved hypothetical protein; Enterobacteria phage SP6 gp23 homolog
orf27	18399..18869	+	471	conserved hypothetical protein; Enterobacteria phage SP6 gp24 homolog
orf28	18978..19925	+	948	conserved hypothetical protein; Enterobacteria phage SP6 gp25 homolog
orf29	19897..20112	+	216	conserved hypothetical protein; Enterobacteria phage SP6 gp25 homolog
orf30	20132..20245	+	114	conserved hypothetical protein; Enterobacteria phage SP6 gp27 homolog
orf31	20242..20703	+	462	putative N-acetyltransferase
orf32	20713..20922	+	210	conserved hypothetical protein; Enterobacteria phage SP6 gp29 homolog
orf33	20924..22474	+	1551	putative head-tail connector
orf34	22474..23394	+	921	putative scaffolding protein
orf35	23471..24676	+	1206	putative major capsid protein
orf36	24731..25468	+	738	putative tail tubular A protein
orf37	25468..27870	+	2403	putative tail tubular B protein
orf38	27870..28598	+	729	putative internal virion protein
orf39	28598..31546	+	2949	conserved hypothetical protein; Enterobacteria phage SP6 gp36 homolog
orf40	31611..34796	+	3186	putative internal virion protein
orf41	34796..35758	+	963	putative tail fiber protein
orf42	35770..35994	+	225	putative holin
orf43	35958..36344	+	387	putative HNH endonuclease
orf44	36385..36684	+	300	putative small terminase subunit
orf45	36684..38582	+	1899	putative large terminase subunit
orf46	38739..39017	+	279	conserved hypothetical protein; Enterobacteria phage SP6 gp42 homolog
orf47	39030..39347	+	318	conserved hypothetical protein; Enterobacteria phage SP6 gp43 homolog
orf48	39340..39684	+	345	conserved hypothetical protein; Enterobacteria phage SP6 gp46 homolog
orf49	39678..39809	+	132	hypothetical protein
orf50	39793..39942	+	150	conserved hypothetical protein; Enterobacteria phage SP6 gp47 homolog
orf51	39935..40156	+	222	conserved hypothetical protein; Enterobacteria phage SP6 gp48 homolog
orf52	40362..40697	+	336	conserved hypothetical protein; Enterobacteria phage K1E hypothetical protein PK1Ep60 homolog
orf53	40764..43199	+	2436	endosialidase; Enterobacteria phage K1 endosialidase homolog
orf54	43177..43326	+	150	conserved hypothetical protein; Enterobacteria phage SP6 gp51 homolog
orf55	43485..43694	+	210	hypothetical protein

Table S3. Summary of ORFs and proteins encoded by phage ACG-M12.

Coordinates	Strand	Length	Gene	Product
528..1025	+	498	orf01	conserved hypothetical protein; Enterobacteria phage RTP protein rtp1 homolog
1025..1309	+	285	orf02	hypothetical protein
1329..1460	+	132	orf03	hypothetical protein
1460..1588	+	129	orf04	hypothetical protein
1585..1815	+	231	orf05	conserved hypothetical protein; Enterobacteria phage RTP hypothetical protein rtp7 homolog
1812..1985	+	174	orf06	conserved hypothetical protein; Enterobacteria phage RB49 conserved hypothetical protein gp55.3 homolog
1982..2116	+	135	orf07	conserverd hypothetical protein; Enterobacteria phage RTP hypothetical protein rtp9 homolog
2191..2400	+	210	orf08	hypothetical protein
2397..2576	+	180	orf09	conserved hypothetical protein; Enterobacteria phage RTP hypothetical protein rtp11 homolog
2626..2763	+	138	orf10	conserved hypothetical protein; Enterobacteria phage RTP hypothetical protein rtp12 homolog
2824..2970	+	147	orf11	hypothetical protein
2970..3212	+	243	orf12	conserved hypothetical protein; Enterobacteria phage RTP hypothetical protein rtp15 homolog
3581..3763	+	183	orf13	conserved hypothetical protein; Enterobacteria phage RTP hypothetical protein rtp17 homolog
3877..4383	+	507	orf14	putative terminase small subunit
4401..5969	+	1569	orf15	putative terminase large subunit
6039..6347	+	309	orf16	conserved hypothetical protein; Escherichia phage phiEB49 gp57 homolog
6428..7684	+	1257	orf17	putative portal protein
7750..8859	+	1110	orf18	putative prohead protease
8872..9381	+	510	orf19	conserved hypothetical protein; Enterobacteria phage RTP hypothetical protein rtp26 homolog
9505..10449	+	945	orf20	conserved hypothetical protein; Enterobacteria phage RTP hypothetical protein rtp27 homolog
10542..10787	+	246	orf21	conserved hypothetical protein; Enterobacteria phage RTP hypothetical protein rtp28 homolog
10829..11230	+	402	orf22	conserved hypothetical protein;Enterobacteria phage RTP hypothetical protein rtp29 homolog
11227..11598	+	372	orf23	conserved hypothetical protein; Enterobacteria phage RTP conserved phage-related protein homolog
11591..12028	+	438	orf24	conserved hypothetical protein; Enterobacteria phage RTP conserved phage-related protein homolog
12018..12419	+	402	orf25	conserved hypothetical protein; Enterobacteria phage RTP hypothetical protein rtp32 homolog
12435..13091	+	657	orf26	putative major tail protein
13201..13467	+	267	orf27	hypothetical protein
13504..13818	+	315	orf28	conserved hypothetical protein; Enterobacteria phage RTP hypothetical protein rtp35 homolog

Table S3. *Cont.*

Coordinates	Strand	Length	Gene	Product
13827..14138	+	312	orf29	conserved hypothetical protein; Enterobacteria phage RTP hypothetical protein rtp36 homolog
14174..17152	+	2979	orf30	putative tail tape-measure protein
17224..17721	+	498	orf31	putative HNH endonuclease
17702..18052	+	351	orf32	putative minor tail protein
18090..18845	+	756	orf33	putative minor tail protein
18921..19274	+	354	orf34	conserved hypothetical protein; Escherichia phage phiEB49 gp37 homolog
20072..20644	+	573	orf36	putative tail assembly protein
20695..24111	+	3417	orf37	putative tail fiber protein
24143..25105	-	963	orf38	conserved hypothetical protein; Enterobacteria phage RTP hypothetical protein rtp44 homolog
25105..25350	-	246	orf39	putative phage lipoprotein
25419..25724	-	306	orf40	conserved hypothetical protein; Enterobacteria phage IME08 hypothetical protein
26060..26245	+	186	orf40A	hypothetical protein
26226..26420	+	195	orf41	conserved hypothetical protein; Enterobacteria phage RTP hypothetical protein rtp46 homolog
26424..26621	+	198	orf42	conserved hypothetical protein; Phage BP-4795hypothetical protein PBV4795_ORF6 homolog
26618..27583	+	966	orf43	putative exodeoxyribonuclease VIII (RecE)
27656..28306	+	651	orf44	putative recombination protein
28354..28815	+	462	orf45	putative single-stranded DNA binding protein
28874..29215	+	342	orf46	conserved hypothetical protein; Escherichia phage phiEB49 gp25 homolog
29255..32074	-	2820	orf47	putative tail fiber
32162..33088	-	927	orf48	putative DNA primase
33144..33650	-	507	orf49	putative transcriptional regulator
33751..35745	+	1995	orf50	putative ATP-dependent helicase
35747..36166	+	420	orf51	conserved hypothetical protein; Enterobacteria phage RTP hypothetical protein rtp54
36243..36440	+	198	orf52	conserved hypothetical protein; Enterobacteria phage RTP hypothetical protein rtp55 homolog
36440..36598	+	159	orf53	hypothetical protein
36598..36819	+	222	orf54	conserved hypothetical protein; Enterobacteria phage RTP hypothetical protein rtp56 homolog
36816..37016	+	201	orf55	conserved hypothetical protein; Escherichia phage phiEB49 gp17 homolog
37013..37207	+	195	orf56	conserved hypothetical protein; Enterobacteria phage RTP hypothetical protein rtp57 homolog
37278..37403	+	126	orf57	conserved hypothetical protein; Enterobacteria phage RTP hypothetical protein rtp58 homolog
37400..37648	+	249	orf58	conserved hypothetical protein; Enterobacteria phage RTP hypothetical protein rtp59 homolog
37651..37902	+	252	orf59	conserved hypothetical protein; Enterobacteria phage RTP hypothetical protein rtp60 homolog
37986..39122	+	1137	orf60	conserved hypothetical protein; hypothetical protein rtp61 Enterobacteria phage RTP homolog
39194..39688	+	495	orf61	putative HNH endonuclease
39765..40238	+	474	orf62	putative polynucleotide kinase/phosphatase
40241..40795	+	555	orf63	conserved hypothetical protein; Enterobacteria phage T1 hypothetical protein T1p62 homolog

Table S3. *Cont.*

Coordinates	Strand	Length	Gene	Product
40864..41040	+	177	orf64	conserved hypothetical protein; Enterobacteria phage T1 hypothetical protein T1p14 homolog
41170..41385	+	216	orf65	putative holin
41386..41871	+	486	orf66	putative endolysin
41847..42236	+	390	orf67	conserved hypothetical protein; Enterobacteria phage RTP hypothetical protein rtp65 homolog
42251..42586	-	336	orf68	conserved hypothetical protein; Enterobacteria phage RTP hypothetical protein rtp66 homolog
42590..44173	-	1584	orf69	conserved hypothetical protein; Enterobacteria phage RTP hypothetical protein rtp67 homolog
44245..44598	-	354	orf70	conserved hypothetical protein; Enterobacteria phage RTP hypothetical protein rtp69 homolog
44600..44761	-	162	orf71	conserved hypothetical protein; Enterobacteria phage RTP hypothetical protein rtp70 homolog
44815..44976	-	162	orf72	conserved hypothetical protein; Enterobacteria phage RTP hypothetical protein rtp72 homolog
44976..45218	-	243	orf73	conserved hypothetical protein; Enterobacteria phage RTP hypothetical protein rtp73 homolog
45211..45402	-	192	orf74	hypothetical protein
45413..45592	-	180	orf75	conserved hypothetical protein; Enterobacteria phage RTP hypothetical protein rtp74 homolog
45589..45756	-	168	orf76	hypothetical protein
45753..45908	-	156	orf77	hypothetical protein
